# Lung cancer prediction in Lambert-Eaton myasthenic syndrome in a prospective cohort

**DOI:** 10.1038/s41598-020-67571-9

**Published:** 2020-06-29

**Authors:** Paul Maddison, Alexander F. Lipka, Paul Gozzard, Girija Sadalage, Philip A. Ambrose, Bethan Lang, Jan J. Verschuuren

**Affiliations:** 1 Department of Neurology, Nottingham University Hospitals NHS Trust, Queen’s Medical Centre, Nottingham, NG7 2UH UK; 2 Department of Neurology, Groene Hart Hospital, Gouda, The Netherlands; 3 Department of Neurology, Sheffield Teaching Hospitals NHS Foundation Trust, Royal Hallamshire Hospital, Sheffield, S10 2JF UK; 4 Division of Neurology, Queens Medical Centre, University of Nottingham, Nottingham University Hospitals NHS Trust, Nottingham, UK; 5 Nuffield Department of Clinical Neurosciences, University of Oxford, West Wing, John Radcliffe Hospital, Oxford, OX3 9DS UK; 6 Department of Neurology, Leiden University Medical Centre, Leiden, The Netherlands

**Keywords:** Neurology, Oncology

## Abstract

To evaluate the Dutch-English Lambert-Eaton Myasthenic Syndrome (LEMS) Tumour Association Prediction (DELTA-P) score in a prospective cohort of patients with newly diagnosed LEMS to assess the clinical validity of this tool in a real-world setting. Clinical features from 87 patients with LEMS, occurring within three months from disease onset, were collated to produce a DELTA-P score for each patient. Lung cancer was detected in 44/87 (51%) LEMS patients. Weight loss ≥ 5%, tobacco use at LEMS onset and age at onset ≥ 50 years were independent predictors for the development of small-cell lung cancer (SCLC) in LEMS patients in multivariable analysis. Median DELTA-P scores were significantly higher in SCLC-LEMS patients (3.5, 95% CI 3 to 4) compared to non-tumour-LEMS (2, 95% CI 1 to 2) (P < 0.0001). Higher DELTA-P scores increased the risk of SCLC stepwise (score 0 = 0%, 1 = 18.8%, 2 = 45%, 3 = 55.5%, 4 = 85.7%, 5 = 87.5%, 6 = 100%). The area under the curve of the receiver operating curve was 82.5% (95% CI 73.9% to 91%). The DELTA-P cancer prediction score, calculated at the time of LEMS diagnosis, is an effective tool for cancer screening in an independent, prospective study setting.

## Introduction

Lambert-Eaton myasthenic syndrome (LEMS) is a presynaptic autoimmune disorder of neuromuscular transmission characterised by proximal muscle weakness and autonomic disturbance^1^. Antibodies to P/Q-type voltage-gated calcium channels (VGCCs) can be detected in over 90% of patients and are responsible for reduction in nerve-evoked release of neurotransmitter at the neuromuscular junction^2,3^. Approximately 60% of LEMS patients have an associated small-cell lung cancer (SCLC), and it is thought that autoantibodies, directed against VGCCs expressed on the tumour surface^4^, cross-react with VGCCs on the presynaptic terminals causing the neurological dysfunction observed. The immune trigger for VGCC antibody production in non-tumour LEMS (NT-LEMS) is unknown.

SCLC is associated with significant morbidity and mortality^5^, and early diagnosis of these lung tumours, in limited stage, is associated with improved cancer survival^6^. Therefore, prompt and intensive screening for SCLC is mandatory in a patient presenting with LEMS^7^. Using this screening protocol, SCLC is detected within 12 months of LEMS diagnosis in over 95% of patients^7^.

We have previously developed and validated a cancer prediction clinical scoring system in Dutch and UK cohorts of LEMS patients from retrospectively collected data (Dutch-English LEMS Tumour Association Prediction, or DELTA-P, score), which discriminated highly between SCLC-LEMS and NT-LEMS, applicable at the time of LEMS diagnosis^8^. The objective of this current study was to evaluate the DELTA-P score for cancer prediction in a new, prospective cohort of patients with newly diagnosed LEMS to assess the clinical validity of this tool in a real-world setting.

## Results

Clinical and demographic details of the 87 prospectively enrolled LEMS patients are shown in Table 1. Forty-three patients (49.4%) had SCLC, and one additional patient (classified in analyses as SCLC-LEMS) had PET/CT evidence of lung cancer in the absence of histological confirmation. Median duration of follow-up in the remaining 43 patients with NT-LEMS was 59 months (range 36 to 100 months). All patients had a pure LEMS phenotype except for two SCLC-LEMS patients who also developed ataxia. The median time between LEMS diagnosis and SCLC confirmation was 0.5 months: the tumour was diagnosed within six months in 91% and within 12 months in 98% of SCLC-LEMS patients. Four SCLC-LEMS patients whose lung tumours were diagnosed more than six months after their LEMS had DELTA-P scores of 1, 2, 4 and 5.

**Table 1:** Clinical and demographic data from prospective LEMS series (within 3 months of symptom onset).

	SCLC-LEMS patients	NT-LEMS patients	Univariable analysis
Number	44	43	
Median age in years (range) at LEMS diagnosis	65 (39–86)	58 (12–83)	P = 0.0082
Aged ≥ 50 years	40/44 (91%)	30/43 (70%)	P = 0.015
Proportion female	28/44 (64%)	26/43 (61%)	P = 0.82
Bulbar/neck weakness	24/44 (55%)	11/43 (26%)	P = 0.008
Sexual impotence^a^	9/44 (21%)	8/43 (19%)	P = 0.52
Male sexual impotence	9/16 (56%)	8/17 (47%)	P = 0.86
Weight loss ≥ 5%	27/44 (61%)	8/43 (19%)	P = 0.0006
Smoking at LEMS onset	29/44 (66%)	7/43 (16%)	P < 0.0001
Karnofsky performance score < 70	23/44 (52%)	10/43 (23%)	P = 0.007
Dry mouth^b^	19/34 (56%)	20/33 (61%)	P = 0.80
Proximal upper limb weakness^c^	20/27 (74%)	16/31 (52%)	P = 0.106
Median survival from LEMS diagnosis (months) (Log rank)	15.6	50	P < 0.0001
Positive P/Q-type VGCC antibodies	42/44 (96%)	36/43 (84%)	P = 0.089
(median titre)	(448 pM)	(209 pM)	(P = 0.0026)

*LEMS* Lambert-Eaton myasthenic syndrome, *SCLC* small-cell lung cancer, *VGCC* voltage-gated calcium channels.

^a^Females scored as not affected

^b^data available on 67/87 LEMS patients;

^c^Data available on 58/87 LEMS patients.

Univariable analysis revealed significant differences between SCLC-LEMS and NT-LEMS for: bulbar symptoms, weight loss ≥ 5% (within 3 months of LEMS onset), tobacco use at LEMS onset, age at onset ≥ 50 years and Karnofsky performance score (Table 1). Weight loss ≥ 5%, tobacco use at LEMS onset and age at onset ≥ 50 years remained significant in multivariable analysis (Table 2).

**Table 2:** Multivariable analysis (logistic regression) analysing risk factors for the development of small-cell lung cancer in patients with Lambert-Eaton myasthenic syndrome (n = 87).

Prognostic factor	Number of events	Odds ratio	LCL	UCL	P value
Bulbar/neck weakness	35	1.514	0.471	4.866	P = 0.486
Male sexual impotence	17	0.946	0.173	5.166	P = 0.949
Weight loss ≥ 5%	35	3.792	1.183	12.158	P = 0.025
Tobacco use at onset	36	8.425	2.607	27.231	P < 0.0001
Age ≥ 50 years	70	9.277	1.3	66.182	P = 0.026
Karnofsky performance < 70	33	2.223	0.617	8.007	P = 0.222

*LCL* lower 95% confidence limit of odds ratio, *UCL* upper 95% confidence limit of odds ratio.

**Figure 1:**
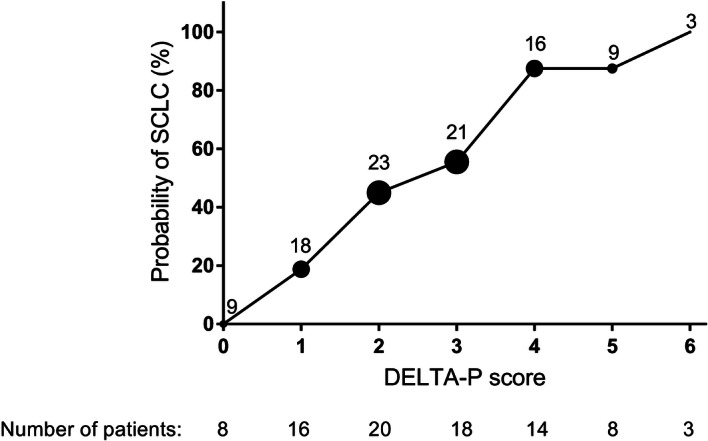
Risk of small-cell lung cancer (SCLC) for each point on the Dutch-English LEMS Tumour Association Prediction (DELTA-P) score in patients with Lambert-Eaton myasthenic syndrome (LEMS) from a prospective cohort (n = 87). Numbers above data points represent the percentage of patients with each score.

Median DELTA-P scores were higher in SCLC-LEMS patients (3.5) compared to NT-LEMS (2) (P < 0.0001). From all 87 patients, a DELTA-P score of 0 or 1 was associated with a null or low risk of developing SCLC (0% and 18.8% respectively); higher DELTA-P scores increased the risk of SCLC stepwise (score 2 = 45%, 3 = 55.5%, 4 = 85.7%, 5 = 87.5%, 6 = 100%)(Fig. 1). The AUC of the ROC curve was 82.5% (95% CI 73.9% to 91%) (Supplementary Fig. 1), with higher AUC values in males (85.3%, 95% CI 71.6% to 98.9%) compared to females (82.01%, 95% CI 70.9% to 93.1%) (P = 0.71). AUC for patients in the Dutch cohort (89.7%, 95% CI 78.6% to 100%) (12/29, 41.4% male; 17/29, 58.6% SCLC) was slightly higher than in the UK cohort (81.1%, 95% CI 70.2% to 92.1%)(21/58, 36.2% male; 27/58, 46.6% SCLC) (P = 0.28). Outcome scores from the second item, “male erectile dysfunction”, showed that this factor was the poorest predictor of SCLC when compared to other components of the DELTA-P score (P = 0.009, Table 3). Eliminating item two to create a 5-point DLTA-P score improved the AUC to 84.5% (95% CI 76.4% to 92.6%) (risk of SCLC: score 0 = 0%, 1 = 18.3%, 2 = 55.6%, 3 = 64.7%, 4 = 81.25%, 5 = 100%), although this was not significantly different from the 6-point DELTA-P score (P = 0.51).

**Table 3:** Individual item performance from the DELTA-P score (prospective study, 87 LEMS patients).

	Scored a 1 correctly for SCLC	Scored a 0 correctly for NO CANCER	Total correct	Scored a 0 incorrectly for SCLC	Scored a 1 incorrectly for NO CANCER	Total incorrect
Item 1 (D)	24 (28%)	32 (37%)	56 (64%)	20 (23%)	11 (13%)	31 (36%)
Item 2 (E)						
All	9 (10%)	35 (40%)	44 (51%)*	35 (40%)	8 (9%)	43 (49%)
Males	9 (27%)	9 (27%)	18 (55%)	7 (21%)	8 (24%)	15 (45%)
Females	0 (0%)	26 (48%)	26 (48%)	28 (52%)	0 (0%)	28 (52%)
Item 3 (L)	27 (31%)	35 (40%)	62 (71%)	17 (20%)	8 (9%)	25 (29%)
Item 4 (T)	29 (33%)	36 (41%)	65 (75%)	15 (17%)	7 (8%)	22 (25%)
Item 5 (A)	42 (48%)	16 (18%)	58 (67%)	1 (1%)	28 (32%)	29 (33%)
Item 6 (P)	23 (26%)	33 (38%)	56 (64%)	21 (24%)	10 (12%)	31 (36%)

*DELTA-P* Dutch-English Lambert-Eaton Myasthenic Syndrome Tumour Association Prediction Score, *SCLC* small-cell lung cancer.

*Item 2 (E) statistically lower than the other five items (P = 0.009).

## Discussion

We have previously developed a highly effective clinical scoring system (DELTA-P) for predicting SCLC development in patients with newly diagnosed LEMS^8^, given that approximately half of all patients with LEMS develop this type of lung cancer. As both the derivation and validation cohorts used to derive the DELTA-P score were analysed retrospectively, we aimed to assess the performance of this score in a new, prospective cohort of LEMS patients.

We found that the DELTA-P score was a very effective tool in predicting SCLC in this new cohort as well, although the score dichotomised less effectively than in the initial study, with poorer discrimination between SCLC-LEMS and NT-LEMS especially in patients with mid-range scores of 2 (45% vs 27% risk) and 3 (55.5% vs 83.9% risk). We found fewer patients with a low risk of SCLC, scoring 0 or 1, compared to the initial DELTA-P study (18.8% vs 35%), but similar numbers of patients at high risk of SCLC, scoring 3, 4, 5 or 6 (53.3% vs 51%). In practice, from our prospective data, this would mean fewer low-risk patients would benefit from a short, two-stage 6 month cancer screening protocol, but similar numbers of high risk patients would require early, intensive screening^7,8^. Of note, almost all SCLC cases were detected using previously published screening guidelines. Three patients with SCLC-LEMS had a DELTA-P score of less than 2. One patient with a DELTA-P score of 1 was diagnosed with SCLC 25 months after LEMS diagnosis highlighting the importance of continued, close clinical vigilance for symptoms and signs of lung cancer even beyond the scheduled tumour screening regimen^7,8^. Although unconfirmed in LEMS, spontaneous tumour regression has been reported in patients with SCLC and Hu-antibody-associated paraneoplastic sensory neuronopathy^9,10^, which could account for delayed tumour detection in other paraneoplastic neurological presentations, perhaps due to an anti-tumour immune response.

It is not unexpected that prognostic scoring systems work less well in subsequent prospective cohorts (real life practice) than in the original datasets^11,12^: derivation cohort analyses that are data-dependent rather than pre-specified give optimistic assessments of future predictive performance in new datasets, and variations in case-mix between study centres can affect score performance, as we found between our NL and UK cohorts.

Differences in the demographic and clinical features (case-mix) of the new cohort compared to the datasets used to define the DELTA-P score may have affected the score’s performance: this new cohort were older (median age 62 years vs 56.8 years, P = 0.011), with more female SCLC patients (64% vs 32.8%, P = 0.002), and a lower frequency of male sexual impotence in SCLC-LEMS patients (20% vs 44%, P < 0.001), although the frequency of cancer occurrence was very similar (51% vs 54.2%)^8^. Nevertheless, the clinical validity of the prognostic DELTA-P score was maintained by studying a similar-sized, relatively large, dataset with a high percentage of outcomes (occurrence of SCLC) per scoring item^13^. The new assessment of DELTA-P performance also benefitted from external validation, where the UK LEMS cohort was assessed by a new assessor (PM) in a new location (Nottingham) compared to the original study.

We found that the performance of the DELTA-P scoring parameter “E”, erectile dysfunction (males), was significantly poorer than the other items, such that removal of this from the DELTA-P score to generate a 5-item classification improved the ROC AUC, albeit non-significantly. In our experience, determining the time of onset of this clinical parameter was difficult, particularly in a cohort where most male patients were aged > 60 years. This would be particularly relevant for patients with co-morbid illness affecting erectile function. Thus, at LEMS diagnosis, if precise data for erectile dysfunction are not available, the prediction of SCLC from a 5-point score would still be accurate.

Using clinically validated data from a large prospective cohort, we have confirmed that the use of the DELTA-P scoring system at LEMS diagnosis is a robust measure of the risk of developing SCLC. However, as a small minority of SCLC-LEMS patients have DELTA-P scores of less than two, the clinical score cannot be used in isolation, and consideration must be made for cancer screening beyond 6 months in patients with low scores (e.g. DELTA-P of 1), particularly for those with co-morbid illness (affecting erectile function) who have previously been smokers. Confirmation of the validity of the DELTA-P scoring system by other international investigators would be of great clinical value.

## Methods

### Patients

Between 2011 and 2017, 87 patients with LEMS were recruited prospectively at the time of neurological diagnosis across two centres in Leiden, Netherlands and Nottingham, UK. All patients were examined by two of the authors (AL, PM). The diagnosis of LEMS was based on characteristic clinical features of proximal limb weakness, attenuated tendon reflexes and autonomic dysfunction, and also either typical neurophysiological findings (low resting compound muscle action potential amplitude in hand muscles, with incremental responses of over 60% after maximal voluntary contraction) or positive P/Q-type voltage gated calcium channel antibodies^1,14–16^. Tumour surveillance with PET/CT imaging was performed as per published guidelines^7^. Briefly, all patients underwent integrated PET/CT imaging at the time of LEMS diagnosis; repeated after three months, then every six months for two years in patients with DELTA-P scores of 3–6; or repeated once after six months in patients with DELTA-P scores of 0–1; or repeated every six months for two years in patients with a DELTA-P score of 2. Beyond this, high clinical vigilance was maintained for symptoms or signs suggestive of SCLC during further clinic visits. All patients with SCLC-LEMS had histological or cytological evidence of SCLC, except one patient without biopsy-proven SCLC whose PET/CT imaging was typical of lung cancer. The remaining patients were classified as NT-LEMS if cancer screening had failed to demonstrate malignancy after three years or more follow-up. Patients without detectable cancer, but follow-up of less than three years were excluded. Demographic details, clinical features and symptoms occurring within three months from disease onset were collated to produce a DELTA-P score for each patient^8^. Written, informed consent was obtained from all patients (Nottingham Research Ethics Committee 04/Q2404/100 and Medical Ethics Committee of the Leiden University Medical Center P13.106). All methods were carried out in accordance with relevant guidelines and regulations and all experimental protocols were approved by an institutional committee (Oxford University, UK, and Leiden University, NL).

### Antibody analysis

Autoantibodies to P/Q-type VGCCs were measured in all patients by either immunoprecipitation of VGCCs extracted from rabbit cerebellum and labelled with 125I-ω-CmTx MVIIC as previously described^3^, or by a radioimmunoassay kit using the same assay principle (from either RSR Ltd., Cardiff, UK, or DLD, Hamburg, Germany) for all Dutch patients. All samples were taken at the time of initial LEMS diagnosis.

### Statistical analysis

Group comparisons were analysed using Fisher’s exact test for frequency distribution, and Mann–Whitney U test for median numerical values. The ability of the DELTA-P score to discriminate between SCLC-LEMS and NT-LEMS was quantified using the area under the curve (AUC) of the receiver operating characteristic (ROC). Area under ROC curve comparisons were made using between-area correlations^17,18^. The covariates for multivariable analysis were selected based on the six individual items of the original DELTA-P score^8^. The probability of developing SCLC for each calculated DELTA-P score was calculated by dividing the number of SCLC-LEMS patients by the total number of LEMS patients with that score (Fig. 1). For individual item performance (Table 3), in each LEMS patient, a correct score of 0 (for each of the six items of the DELTA-P score, in turn) meant the patient had no SCLC; a correct score of 1 meant the patient had SCLC; an incorrect score of 0 meant the patient had SCLC; an incorrect score of 1 meant the patient had no SCLC.

## Supplementary information

Supplementary information

## Data Availability

The authors are happy to make materials, data and associated protocols promptly available to readers without undue qualifications in material transfer agreements.
