# Supplementary material for: Lung cancer prediction in Lambert-Eaton myasthenic syndrome in a prospective cohort
Source: Sci Rep. 2020 Jun 29;10:10546. doi: 10.1038/s41598-020-67571-9 (PMC7324357; doi:10.1038/s41598-020-67571-9)
Supplement: Supplementary file 1 — Supplementary information [file 41598_2020_67571_MOESM1_ESM.docx]

**Cancer prediction in Lambert-Eaton myasthenic syndrome in a prospective cohort**

Paul Maddison, Alexander F Lipka, Paul Gozzard, Girija Sadalage, Philip A Ambrose, Bethan Lang, Jan J Verschuuren

**Supplementary data**

**Cancer prediction in Lambert-Eaton myasthenic syndrome in a prospective cohort**

**Supplementary figure 1.** Receiver operating characteristic curve for DELTA-P scores in the prospective LEMS cohort (n=87)(AUC=82.5%).

**Dutch IRB approved protocol**

**Lambert-Eaton myasthenic syndrome:
Clinical characteristics,
pathogenesis and
tumour association**

**(23 May 2013)**

**PROTOCOL TITLE**

**Lambert-Eaton myasthenic syndrome: Clinical characteristics, pathogenesis and tumour association**

| **Protocol ID** | **LEMS02** |
| --- | --- |
| **Short title** | **LEMS and SCLC** |
| **Version** | **1.0** |
| **Date** | **23 May 2013** |
| **Coordinating investigator/project leader** | **Prof. J.J.G.M. Verschuuren, MD, PhD Neuromuscular Diseases**  **Dept. Neurology Leiden University Medical Center (LUMC) P.O. Box 9600 2300 RC Leiden The Netherlands Tel 071-5262197 Fax 071-5248253 E-mail:** [**J.J.G.M.Verschuuren@lumc.nl**](mailto:J.J.G.M.Verschuuren@lumc.nl) |
| **Principal investigator** | **Prof. J.J.G.M. Verschuuren, MD, PhD Neuromuscular Diseases**  **Dept. Neurology Leiden University Medical Center (LUMC) P.O. Box 9600 2300 RC Leiden The Netherlands Tel 071-5262197 Fax 071-5248253 E-mail:** [**J.J.G.M.Verschuuren@lumc.nl**](mailto:J.J.G.M.Verschuuren@lumc.nl) |
| **Sponsor** | **LUMC, Leiden** |
| **Independent physician(s)** | **Mw. Dr. M.J.H. Wermer, dept. neurology,  LUMC, tel. 071-5262134** |
| **Laboratory sites** | **Prof. S.H. van der Burg, MD, PhD Dept. Clinical oncology**  **LUMC, tel. 071-5263464** |

**PROTOCOL SIGNATURE SHEET**

| **Name** | **Signature** | **Date** |
| --- | --- | --- |
| **For non-commercial research,**  **Head of Department:**  Prof. dr. R.A.C. Roos,  neurologist dept. neurology, LUMC |  |  |
| **Coordinating Investigator/Project leader/Principal Investigator:**  Prof. dr. J.J.G.M. Verschuuren,  neurologist  dept. neurology, LUMC  **Investigator:** drs. A.F. Lipka  Dept. of neurology  LUMC  a.f.lipka@lumc.nl |  |  |
|  |  |  |

**TABLE OF CONTENTS**

1. INTRODUCTION AND RATIONALE 7

2. OBJECTIVES 11

3. STUDY DESIGN 12

4. STUDY POPULATION 13

4.1 Population (base) 13

4.2 Inclusion criteria 13

4.3 Exclusion criteria 13

5. METHODS 14

5.1 Study parameters/endpoints 14

5.2 Study procedures 16

5.2.1 Blood sample 16

5.2.2 Electromyography 17

5.2.3 Tumour tissue 17

5.2.4 Imaging studies 17

5.2.5 Analysis of humoral and cellular immune response 18

5.3 Withdrawal of individual subjects 20

6. STATISTICAL ANALYSIS 20

7. ETHICAL CONSIDERATIONS 21

7.1 Regulation statement 21

7.2 Recruitment and consent 21

7.3 Benefits and risks assessment, group relatedness 21

7.4 Compensation for injury 22

7.5 Incentives (if applicable) 22

8. ADMINISTRATIVE ASPECTS AND PUBLICATION 23

8.1 Handling and storage of data and documents 23

8.2 Amendments 23

8.3 Annual progress report 23

8.4 End of study report 23

8.5 Public disclosure and publication policy 23

9. REFERENCES 24

**LIST OF ABBREVIATIONS AND RELEVANT DEFINITIONS**

| **CCMO** | **Central Committee on Research Involving Human Subjects; in Dutch: Centrale Commissie Mensgebonden Onderzoek** |
| --- | --- |
| **CMAP** | **Compound Muscle Action Potential** |
| **DC** | **Dendritic Cells** |
| **EMG** | **Electromyography** |
| **HLA** | **Human Leukocyte Antigen** |
| **KIR** | **Killer immunoglobulin-like receptor** |
| **LEMS** | **Lambert-Eaton Myasthenic Syndrome** |
| **LUMC** | **Leiden University Medical Center** |
| **METC** | **Medical research ethics committee (MREC); in Dutch: medisch ethische toetsing commissie (METC)** |
| **MG** | **Myasthenia Gravis** |
| **PBMC** | **Peripheral Blood Mononuclear Cell** |
| **SCLC** | **Small Cell Lung Cancer** |
| **Sponsor** | **The sponsor is the party that commissions the organisation or performance of the research, for example a pharmaceutical**  **company, academic hospital, scientific organisation or investigator. A party that provides funding for a study but does not commission it is not regarded as the sponsor, but referred to as a subsidising party.** |
| **VGCC** | **Voltage-Gated Calcium Channels** |
| **WMO** | **Medical Research Involving Human Subjects Act (in Dutch: Wet Medisch-wetenschappelijk Onderzoek met Mensen** |

**SUMMARY**

**Rationale:** A detailed description of clinical characteristics and disease course of patients with Lambert-Eaton myasthenic syndrome (LEMS) will enable earlier diagnosis and treatment of this rare, disabling, but well-treatable, neuromuscular disorder and the associated tumour. This study will provide detailed knowledge about the immune response against voltage-gated calcium channels and the small cell lung cancer (SCLC) in patients with LEMS. This is essential for the development of future therapeutical interventions or immune-mediated therapies.

**Objective**:

- To study the clinical characteristics and disease course of patients with LEMS with or without SCLC.
- To identify the characteristics of the humoral or cellular immune response that are associated with the prolonged survival of patients with SCLC and LEMS.

**Study design:** Single center prospective study

**Study population:** Patients with Lambert-Eaton myasthenic syndrome or SCLC.

**Main study parameters/endpoints:** Description of epidemiology, clinical characteristics and disease course in LEMS patients. Prediction of presence of an underlying SCLC in LEMS patients. Comparison of the humoral and cellular immune response in LEMS patients and SCLC patients with and without LEMS.

**Nature and extent of the burden and risks associated with participation, benefit and group relatedness:** Patient burden and risk in this study will be minimal and mostly almost completely limited to the period shortly after inclusion. If a patient participates in all elements of the study this will include:

- detailed history and physical examination

- electromyogram to determine the severity of the LEMS

- blood donation. We will draw 90 mL at inclusion and up to 50 mL at follow-up for selected patients with SCLC.

Personal benefit would be the contact with the clinical researchers experienced in the treatment of this rare disorder, with the opportunity to be completely informed on all aspects of the disease and get detailed therapeutical advice. At a group level the results will contribute to improved understanding of the pathogenesis, and possibly improved therapies.

# INTRODUCTION AND RATIONALE

LEMS is an antibody-mediated autoimmune disorder characterised by proximal muscle weakness, loss of tendon reflexes and autonomic dysfunction.

*Clinical features*

It can occur at all ages and affects both men and women. LEMS is associated with pathogenic antibodies against the presynaptic P/Q-type voltage-gated calcium channel (VGCC) in 90% of patients. In about 50-60% of patients small cell lung cancer (SCLC) is detected.^1^

LEMS usually presents with progressive proximal leg weakness, which occurs in virtually all patients during the course of the disease. As in myasthenia gravis (MG), weakness usually progresses to other muscle groups. These include the arms, feet and oculobulbar muscles causing ptosis, diplopia and dysarthria. However, in LEMS weakness generally spreads in caudocranial direction, while in myasthenia gravis it usually spreads in the opposite direction.^2^ In contrast to MG, symptoms of autonomic dysfunction occur in 80-90% and include dry mouth and eyes, erectile dysfunction, constipation and blurred vision. Although rare, respiratory failure can occur and is described as presenting symptom in single cases.

*Diagnosis of LEMS*

Diagnosis of LEMS usually precedes diagnosis of SCLC by several months, thus prompting vigorous tumour screening.^3^ The presence of an underlying SCLC causing LEMS is of uttermost importance and therefore discriminating SCLC-related LEMS from non-tumour (NT)-LEMS as well. Although comparable at first sight, SCLC-LEMS is generally a more rapidly progressive and severe disease, with weakness spreading more quickly.

*Pathogenesis*

The pathogenesis of LEMS is known in detail. IgG antibodies to presynaptic P/Q-type voltage gated calcium channels cause muscle weakness and autonomic dysfunction by interfering with Ca2+-dependent neurotransmitter release.^1, 4^ The target of these antibodies, the P/Q-type VGCC, is present at the neuromuscular junction as well as in SCLC tumour cells.^5^ These antibodies can block Ca2+ influx through voltage-gated calcium channels in SCLC cell lines.^4^ It is likely that that an immune reaction against antigenic determinants on the tumour's surface triggers autoantibody production, and these antibodies cross-react with VGCC on the nerve terminals and cause neurological disease. The detailed insight into the pathophysiology and interaction between LEMS and SCLC make LEMS an excellent candidate to study mechanisms of both general autoimmunity and tumour immunology.

*LEMS and small cell lung cancer*

Lung cancer is the number one cause of cancer-related death in both women and men. Small cell lung cancer (SCLC) accounts for approximately 13-16% of pulmonary tumours and is an aggressive disease with a median survival of only 10 months.^6-8^ Only modest improvements have been seen in survival over the last 30 years.^6^ In the Netherlands each year about 1700 patients are diagnosed with a SCLC. It is a very immunogenic tumour, frequently eliciting antibodies against tumour antigens. Many of the SCLC-associated antigens are also present in the nervous system, due to the neuro-endocrine origins of SCLC. Several tumour related neurological autoimmune syndromes have been described in SCLC patients. The best defined of these paraneoplastic disorders are the Lambert-Eaton myasthenic syndrome (LEMS) and the anti-Hu syndrome.

A profound effect on survival has been observed in SCLC patients with a paraneoplastic neurological disorder. Supporting a role for an effective anti-tumour immune response are reports of spontaneous remission of a proven lung tumour.^9, 10^ The most impressive prolonged survival has been observed in patients with LEMS. This effect has been shown both in our cohort as well as an independent British cohort. Three studies report a significantly prolonged median survival of 17, 20 or 24 months, compared to 10 months in the control groups of SCLC patients.^11-13^ In our cohort, three year survival was improved from 2% to 33%.^14^ This suggests a role for the immune response in controlling the tumour growth. Besides an antibody response, SCLC is an important immunological target for a cellular immune response as well. Previous studies have described the role of tumour-infiltrating lymphocytes and macrophages in immune responses to several tumour types.^15-17^ In SCLC, abundant presence of these cell types infiltrating the tumour has been associated with a favourable disease course.^15^ Overall, both cellular and humoral immunity seem to differ in SCLC patients with and without paraneoplastic syndromes, reflecting a more tumour-rejecting type of immune response in patients with paraneoplastic neurological disease.^15, 18^ However, the mechanism by which these immune responses can influence tumour growth remain unclear.

*Role for genetic variation*

Genetic variation in several genes has been associated with variation in susceptibility to and survival of SCLC in several studies.^19, 20^ Gene groups responsible for immune regulation such as human leukocyte antigen (HLA) and Killer immunoglobulin-like receptor (KIR) have also been shown to differ in occurrence in both SCLC as well as the associated anti-Hu paraneoplastic syndrome.^21, 22^ Future candidate gene and association studies could further indicate the role for specific gene markers which determine susceptibility and prognosis.

*Treatment*

The treatment consists of symptomatic treatment (3,4-diaminopyridine or pyridostigmine), prompt tumour treatment or immunosuppression, including prednisolone, azathioprine or cyclosporine.^23^ Current treatment is able to control signs and symptoms, but often at the cost of more or less severe side-effects of immunosuppressive or lung cancer treatment. The long-term prognosis of non-tumour LEMS regarding life expectancy is good, in contrast to the LEMS patients with SCLC. The presence of LEMS in a patient with SCLC improves survival, but as discussed above only one third of the patients survives beyond 3 years.^14^

Further insight into immune response and improved tumour treatment would be very helpful.

This study protocol is an extension of the previous protocol titled “Klinische manifestaties, pathogenese en behandeling van het Lambert-Eaton myastheen syndroom” (protocol number P181/98) which has previously been approved by the METC. Our research group has collected and characterized 120 patients with LEMS, which now is the largest well-defined cohort of LEMS patients worldwide.

*Preliminary results*

Our studies over the last years have focussed on the clinical manifestations and treatment as well as pathogenesis of LEMS and interaction with SCLC. These have led to several publications in high-ranking journals, like Journal of Clinical Oncology or Lancet Neurology.^1, 24, 25^

We were first to describe antibodies to the VGCC-beta subunit in patients with LEMS and detected the HLA association of LEMS.^26, 27^ Non-tumour LEMS is strongly associated with the highly conserved HLA-8.1 haplotype, indicating a hereditary genuine risk for developing an autoimmune disease. SCLC-LEMS patients carry HLA-types not different from the general population, suggesting that SCLC tumour cells actually elicit the autoimmune reaction irrespective of the underlying HLA haplotype.

In collaboration with all university hospitals and several larger non-university hospitals in the Netherlands we now have collected information on 120 patients with LEMS, with or without SCLC. Our recent projects on LEMS have resulted in a detailed description of the epidemiology of LEMS.^1, 28^ We have described the clinical manifestations in multiple publications.^2, 29, 30^ We have conducted a randomized, double-blind, crossover study showing the efficacy of 3,4-diaminopyridine compared to pyridostigmine and placebo.^31^

About half of the Dutch LEMS patients develop a SCLC. We have shown a prolonged survival in SCLC patients with LEMS compared to patients with SCLC alone.^11, 14^ We also evaluated the screening for SCLC in patients with LEMS and showed that a SCLC can be found in 96% of SCLC-LEMS patients within a year of the diagnosis of LEMS. We demonstrated that a CT-thorax is superior to X-thorax to detect a SCLC in LEMS (92% vs. 51%).^3^ Detailed analysis of clinical and demographic characteristics from two independent patient cohorts has led us to develop the DELTA-P score.^25^ This prediction model has provided for a simple clinical tool to indicate the presence of SCLC early in the course of the disease and can be used to guide tumour screening in individual patients.

In collaboration with Barcelona, we described SOX1 antibodies to be specific for LEMS patients with SCLC.^32, 33^ To further study the relationship with SCLC, a collaboration with the laboratory of prof Sjoerd van den Burg has been started. His lab has extensive experience with several in vitro tumour models and the study of T cells, macrophages, cytokines and their interactions.^34-36^ This setup is readily available to be applied to SCLC cell lines and patient derived mononuclear blood cells. Subsets of macrophages and T cells in several solid tumours were defined that correlate with different anti-tumour immune responses.

*Rationale*

We will continue to study clinical characteristics and disease course of LEMS patients in the Netherlands. Further characterisation of this rare disease will increase recognition of corresponding symptoms, thus enabling earlier diagnosis.

This study will also provide detailed knowledge about the immune response against SCLC in LEMS patients. We will classify SCLC patients with and without LEMS according to immunological profile and give insight into the cellular immune response. This could elucidate pathways to future immune-based therapeutic strategies aimed at SCLC.

In addition, analysis of the anti-tumour immune response will aid our understanding of the generation of an autoimmune neurological disease from this immune reaction. Especially in LEMS, the strong tumour association and detailed insight into the pathophysiology make this disease an ideal candidate to study mechanisms of general autoimmunity as well as tumour immunology.

# OBJECTIVES

*Main objective:*

To understand the pathophysiology of the immune response against VGCC in tumour- and non-tumour related LEMS.

*Specific objectives:*

1. To describe in detail the epidemiology, clinical characteristics (including imaging studies) and disease course of the Lambert-Eaton myasthenic syndrome with or without SCLC.
2. To prospectively test the ability of the DELTA-P score to predict the presence of an underlying SCLC in LEMS patients.
3. To study biomarkers in the peripheral blood of patients with LEMS, LEMS and SCLC, or SCLC only.
4. To study the humoral and cellular aspects of the tumour immune response in patients with SCLC and LEMS.

# STUDY DESIGN

This study will be a single center prospective study. An overview of procedures is further detailed in section 5.

Duration: Starting spring 2013

Setting: The study will be conducted at LUMC, Leiden, the Netherlands. Our neuromuscular outpatient clinic has longstanding expertise in diagnosing and treating LEMS patients. Patients with this diagnosis are referred to our center on a regular basis for second opinion. All academic centers in the Netherlands will also cooperate in referring newly diagnosed LEMS patients

We will include 50 consecutive LEMS-negative SCLC patients diagnosed in the LUMC as a control group for immunological studies.

# STUDY POPULATION

## Population (base)

We will aim to include all patients diagnosed with Lambert-Eaton myasthenic syndrome in the Netherlands.

Inclusion of patients with LEMS can occur by 3 different methods.

1. Newly diagnosed LEMS patients in the neuromuscular outpatient clinic of the LUMC
2. LEMS patients diagnosed elsewhere. All academic centres and a large number of neurologists from peripheral centres in the Netherlands have been cooperating over the years in referring newly diagnosed LEMS patients for second opinion and therapy advice.
3. All patients tested positive for VGCC antibodies in the LUMC (laboratory dr. ir. B.E.P.B. Ballieux) and the Erasmus MC (laboratory prof. dr. H. Hooijkaas). We will contact the physicians of patients positive for VGCC antibodies and ask them to inform their patients of our study. A positive test VGCC antibodies is highly predictive for the presence of clinically relevant LEMS. (attachment E3.1, E3.2).

Inclusion of SCLC patients

1. The department of pulmonology will refer all patients diagnosed with SCLC.

For some objectives (imaging studies, biopsies), retrospective patients in the LUMC will be included.

## Inclusion criteria

*Diagnosis of LEMS*
Criterion 1 and 2 or 1 and 3 have to be fulfilled

1. Clinical features consistent with LEMS (proximal muscle weakness, reduced tendon reflexes, autonomic symptoms)
2. Abnormalities upon repetitive nerve stimulation (decrement of CMAP amplitude and/or increment after voluntary contraction or high frequency stimulation)
3. Presence of VGCC antibodies

*Diagnosis of SCLC*

Definitive diagnosis of SCLC based on pathology results (either cytology or histology).

## Exclusion criteria

- Age < 18 years
- Unable to give informed consent

# METHODS

## Study parameters/endpoints

Parameters/endpoints for each of the objectives stated in chapter 3:

1. Epidemiology, clinical characteristics and disease course of LEMS with/without SCLC

*Introduction*

Many of our previous studies have focussed on clinical characteristics of LEMS patients. Based on the previous years, we expect to include 4-6 LEMS patients per year. For most clinical characteristics and the disease course, we will not define specific endpoints in advance.

We will specifically focus on lung imaging studies to determine tumour size and growth upon diagnosis in SCLC-LEMS patients compared to SCLC only. A prolonged survival has been observed in SCLC-LEMS patients, as well as increased tumour infiltration by macrophages.^16^ Lung tumours in LEMS are also frequently present in mediastinal lymph nodes only. Imaging studies in these patients might therefore show a difference in tumour size and growth as well as distribution.

*Hypothesis*

Lung tumours in SCLC-LEMS patients are smaller on average and grow at a slower rate compared to tumours in SCLC patients without LEMS.

*Endpoints*

Tumour size and growth rate upon follow-up.

Distribution of metastases.

1. To prospectively test the ability of the DELTA-P score to predict the presence of an underlying SCLC in LEMS patients.

*Introduction*

Detailed analysis of clinical and demographic characteristics from two independent patient cohorts has led us to develop the DELTA-P score.^25^ This prediction model has provided for a simple clinical tool to indicate the presence of SCLC early in the course of the disease and can be used to guide tumour screening in individual patients. This model has not yet been investigated prospectively.

*Hypothesis*

The DELTA-P score accurately predicts presence of SCLC in newly diagnosed LEMS patients.

*Endpoints*

Clinical characteristics necessary for DELTA-P score.

Diagnosis of SCLC upon follow-up.

1. Biomarkers in the peripheral blood of patients with LEMS, SCLC-LEMS, or SCLC only.

*Introduction*

Multiple biomarkers for LEMS have been studied in our cohort. We have previously identified SOX antibodies as specific serological marker for SCLC-LEMS.^24, 33^ Antibodies against the intracellular Hu protein are also found in SCLC patients with and without LEMS. Additionally, HLA-B8-DR3 was identified as a biomarker for LEMS without associated tumour.^14, 27^

*Hypothesis*

Multiple biomarkers including auto-antibodies and HLA type are present in LEMS and are associated with specific disease subgroups.

*Parameters*

We will test for Hu and SOX antibodies as well as HLA type.

*Future objectives:*

It is likely that in the future other biomarkers will become available. Currently, we are collaborating with prof. dr. S.M. van der Maarel and dr. P.A.C. ‘t Hoen (Dept. of Human Genetics) to search for new biomarkers. We expect that our study will identify patients with prolonged tumour survival or a favourable anti-tumour immune response. For these patients, genetic polymorphisms will be valuable for our understanding of the immune mechanism. The unfortunate poor survival of this patient group makes it impossible to ask for DNA samples to be taken later on during the study. Thus, because of the nature of this prospective study, combining detailed clinical and immunological data, we will store DNA for analysis in future studies.

1. Humoral and cellular aspects of the tumour immune response in SCLC-LEMS

*Introduction*

SCLC is a very immunogenic tumour, leading to more frequent occurrence of paraneoplastic syndromes compared to other tumours.^5^ The prolonged tumour survival associated with some of these paraneoplastic syndromes suggests a functional immune response against both tumour and healthy tissue. However, the pathways by which the immune system can control tumour growth remain unclear. We will study multiple possible pathways that could lead to a more tumour-rejecting type of immune response.

*Hypothesis*

- SCLC biopsies in LEMS patients show an increase in tumour infiltration by lymphocytes and macrophages.
- An anti-tumour effect of serum VGCC antibodies and related cellular immune responses can be detected *in vitro*.
- Specific T cells against tumour-related antigens, such as VGCC, are present in serum of LEMS patients.

*Parameters*

- Tumour infiltration of SCLC by lymphocytes and macrophages (+- LEMS)
- Anti-tumour effect of serum VGCC antibodies and related macrophage and adaptive response in vitro
- Presence of specific T cells against tumour-related antigens

## Study procedures

Available clinical information will be collected from all patients both at inclusion and at follow-up. After inclusion, we will aim to investigate all patients in the outpatient clinic of the Department of Neurology, LUMC. In our experience, some patients with both SCLC and LEMS will not be able or willing to visit the LUMC because of their physical condition, especially after starting chemo- and radiotherapy. In order to maximize participation, we will still ask their consent for obtaining medical correspondence and for a researcher to visit and see them in their local hospital.

### Blood sample

Each patient will be asked to donate blood. It will concur with the regular blood sample drawn at diagnosis.

We will draw 90 ml of blood additionally:

-70 ml to isolate serum, test for antibodies and peripheral blood mononuclear cells

-20 ml to isolate and store DNA

For patients with SCLC, we will draw up to 50 ml of blood a second time at follow-up for immunophenotyping.

The samples will be handled in Leiden to extract DNA (Laboratory for Diagnostic Genome Analysis, prof.dr. E. Bakker), to obtain serum and to isolate cells that will be frozen and stored (laboratory of Clinical Oncology, prof. dr. S.H. van der Burg).

### Electromyography

Patients with clinical signs or symptoms of LEMS will all be seen by a neurologist from the LUMC. They will be asked to have an electromyographical study (EMG). This forms a very sensitive test for LEMS. For LEMS the amplitude of the compound muscle action potential (CMAP) and the amount of decrement or increment will be determined by repetitive nerve stimulation and registration from the abductor digiti minimi, nasalis and trapezius muscles. This investigation is part of regular care for patients with LEMS.

### Tumour tissue

During the diagnostic workup some SCLC patients will have had a tumour biopsy. We will ask these patients for permission to obtain tumour samples to study the characteristics of the cellular infiltrates. Samples will be collected from SCLC patients and from SCLC-LEMS patients. All samples will be obtained for diagnostic purposes, thus before the start of chemotherapy. All samples will be coded (SCLC+patient group+number in order of inclusion, e.g. SCLC_LEMS_001), which can be traced back to patient information only by the investigators (SL and JV). Any clinically relevant findings will be discussed with the treating pulmonologist.

We will also try to obtain tumour tissue retrospectively. In our previous study (protocol number P181/98) we have collected clinical data of multiple patients with both SCLC and LEMS. These patients have given their permission (either written or verbal) to collect their clinical data from their previous treating neurologist. The vast majority of these patients will be deceased at the start of this protocol. It is not desirable to burden their relatives with this explicit permission for obtaining tumour tissue. Also, use of tumour tissue for research purpose is not harmful for patients or their relatives. For these reasons, we will not obtain specific permission for obtaining these tumour samples from relatives. Instead, we will aim to obtain tumour tissue by contacting the previously treating neurologist, (attachments E3.3-E3.4).

### Imaging studies

For patients with SCLC we will ask for permission to collect their previous lung imaging studies. This will allow us to compare size and distribution of the lung tumour for patients with and without LEMS. As for tumour tissue, we have collected clinical data of multiple SCLC-LEMS patients. For the same reasons stated in the previous paragraph, we will not obtain specific permission for obtaining imaging studies of patients who are already deceased. Instead we will request these imaging studies directly, stating this procedure is supported by the METC.

### Analysis of humoral and cellular immune response

*Mechanism of tumour immune response*

Tumour infiltration by lymphocytes and macrophages has been investigated in detail in several types of cancer including SCLC and seems to be associated with survival advantage.^15, 17, 37^ SCLC is a very immunogenic tumour, leading to more frequent occurrence of paraneoplastic syndromes compared to other tumours.^5^ The prolonged tumour survival associated with some of these paraneoplastic syndromes suggests a functional immune response against both tumour and healthy tissue. However, the pathways by which the immune system can control tumour growth remain unclear. The frequent occurrence of onconeuronal antibodies without the corresponding clinical syndrome is not related to a survival advantage in SCLC patients, suggesting that the antibody response is required but not sufficient to cause the clinical syndrome, including an effective anti-tumour response.^11, 38^ We will study multiple possible pathways that could lead to a more tumour-rejecting type of immune response. Also, we will search for other contributing immune responses, e.g. tumour infiltration, that can influence prognosis in SCLC patients.

*a). What type of macrophages are present in LEMS+ SCLC?*

Tumour-promoting M2 type macrophages are identified via their expression of CD163. Recently we showed that these cells can be easily identified via staining for CD68 (all types of macrophages) and the absence (M1 type) or presence (M2 type) of CD163.^34^

We will stain SCLC tumour sections from patients with and without LEMS and VGCC antibodies for different antigen-presenting cell markers and quantify these cells per square mm tumour. Quantification can be automatically done.^34^ This will allow us to assess what type of function the macrophages may have within the tumours of LEMS+ patients.

We will also determine whether predominance of a macrophage subtype can predict survival in all SCLC patients.

*b). Tumour-infiltrating lymphocytes*

To assess if lymphocyte infiltration (especially B-cells) of SCLC is related to better survival for SCLC patients with Hu, SOX and VGCC antibodies and SCLC patients without PNS, we will stain tumour sections for CD20 (B cells), and CD3 (T cells) to quantify and determine their relation with disease-free and overall survival. If T cells play a role we will stain a number of tumours for CD8, CD4 and Foxp3 in order to gain more information about the type of T cell infiltrate (CD8; cytotoxic T cells, CD4; T-helper cells, CD4+Foxp3+; regulatory T cells).

*c). Do VGCC antibodies mediate an anti-tumour effect?*

High titres of VGCC-specific IgG antibodies are readily detected in the serum of patients. There are a number of SCLC cell lines available (e.g. NCI-H146, NCI-H345), which display functionally active P/Q type of VGCC.^39^ Microscopy studies suggest that VGCCs are desorganized and become clustered on the cells of LEMS patients resulting in lowered signalling function, however this would allow VGCC-specific antibodies to bind more easily and to function as targets for activated macrophages.^40^

- To assess whether VGCC-antibodies can kill tumour cells via complement binding, we will incubate P/Q type VGCC positive and negative SCLC with serum of VGCC+-SCLC patients and study direct tumour kill.^41, 42^ As a control we will also use serum of VGCC- SCLC patients, as well as heat-inactivated serum to show the effect is complement-mediated.

- To assess whether VGCC-specific antibodies can induce antibody dependent cellular cytotoxicity we will incubate the cell lines with patient-derived serum, add activated M1 or M2 macrophages and study tumour kill.^41, 43^

*d). Can VGCC antibodies stimulate the adaptive anti-tumour response?*

Recently we showed that the binding of antibodies to tumour cells can directly influence the local tumour environment by binding to the Fc-receptors present at the surface of macrophages which – through their fixed state on tumour cells – will result in the activation of the macrophage. Dependent on the abundance of the type of macrophage this may result in a tumour-rejecting or tumour-promoting environment.^36^ Based on the expression of the P/Q-type VGCC at the surface of tumour cells, bound VGCC antibodies are also likely to induce macrophage activation.

We will add VGCC Ab+ and VGCC Ab- serum to tumour cell lines and incubate them with in vitro produced M1 and M2 macrophages as well as dendritic cells (DC). The macrophages and DC will be studied for the typical surface markers associated with activation, as well as for the production of cytokines associated with tumour-rejection (IL12p70) or tumour-promotion (IL-10, IL-6).

*e). Do SCLC patients display specific CD4+ T-cells against tumour-related antigens?*

The presence of VGCC, Hu and SOX IgG antibodies indicate that T-helper dependent antibody isotype switching has occurred, and as a consequence that patients would have mounted an antigen-specific CD4+ T cell response. While such an auto-immune response is likely to be controlled by regulatory T cells (Tregs), patients with PNS, including LEMS, show lower levels of Tregs. We have produced recombinant proteins of the different VGCC sub-units. BMC isolated from LEMS+ and LEMS- patients with SCLC will be stimulated with autologous recombinant protein pulsed monocytes and tested by proliferation assays, cytokine ELISA and multiparameter flow cytometry in order to determine the presence, specificity and type of VGCC-specific T cells as described before by us.^44^

*f). Do VGCC antibodies lead to improved tumour-immunity via opsonization of tumour antigens?*

SCLC tumour cells expressing P/Q-type VGCC will be incubated with or without VGCC antibodies (or VGCC-Ab+patient serum) and co-cultured with monocyte-derived M1 macrophages or dendritic cells from patients’ peripheral blood mononuclear cells (PBMC). Autologous (patient-derived) PBMC will be added and the difference in stimulation of T cells will be followed through the expression of early and late activation markers as well as the production of cytokines.

*g). Do SCLC patients with and without LEMS differ in cellular immune response?*

We will study peripheral blood mononuclear cells using immunophenotyping by flow cytometry. We will compare LEMS+, LEMS- SCLC patients and LEMS patients without associated tumour. In SCLC patients, we will ask patients to donate blood upon follow-up as well, concurring with a regular blood sample. This will allow us to study the cellular immune response before and after chemotherapy.

## Withdrawal of individual subjects

Subjects can leave the study at any time for any reason if they wish to do so without any consequences. The investigator can decide to withdraw a subject from the study for urgent medical reasons.

# STATISTICAL ANALYSIS

We will establish a database (MS Access) for clinical characteristics and lab results. Statistical analysis will be carried out by SPSS (SPSS 20.0, Chicago, IL, USA) and / or GraphPad Prism 5.01 (GraphPad Software Inc, La Jolla, CA, USA).

# ETHICAL CONSIDERATIONS

## Regulation statement

The study will be conducted in compliance with the protocol, the ethical principles that have their origin in the Declaration of Helsinki (Version 9, amended by the 59^th^ WMA general Assembly, Seoul, October 2008) and in accordance with the Medical Research Involving Human Subjects Act (WMO).

## Recruitment and consent

Patients with LEMS will be included by the investigators at the neuromuscular outpatient clinic in the LUMC. They will be informed of the study both verbally and using the patient information letter. Subjects will usually be given up to 24 hours to consider their decision; however this also depends on the planned moment of regular blood sampling. Patient information letter and informed consent form for LEMS patients are attached (E1.1 and E2.1).

Patients identified by a positive test for VGCC antibodies will be informed by mail via their treating neurologist (attachment E3.1-E3.2). They will be included if they return the informed consent form.. They can also decide to participate only in a part of this study, e.g. by only donating blood, if they are unable or willing to visit the LUMC outpatient clinic (paragraph 5.2).

Patients with SCLC will be included by their pulmonologist after pathological confirmation of the diagnosis. Any questions about the study can be directed either at their pulmonologist or at the investigators at a later moment. Subjects will usually be given up to 24 hours to consider their decision; however this depends on the planned moment of regular blood sampling. Patient information letter and informed consent form specifically for SCLC patients are attached (E1.2 and E2.2).

## Benefits and risks assessment, group relatedness

Patient burden and risk in this study will be minimal and mostly almost completely limited to the period shortly after inclusion. If a patient participates in all elements of the study this will include:

- detailed history and physical examination

- electromyogram to determine the severity of the LEMS

- each patient will be asked to donate blood. We will draw 90 mL at inclusion and up to 50 mL at follow-up for selected patients with SCLC. This will concur with regular blood samples.

Personal benefit would be the contact with the clinical researchers experienced in the treatment of this rare disorder, with the opportunity to be completely informed on all aspects of the disease and get detailed therapeutical advice.

At a group level the results will contribute to improved understanding of the pathogenesis, more accurate diagnosis and prognosis, and possibly improved therapies.

## Compensation for injury

The sponsor/investigator has a liability insurance which is in accordance with article 7, subsection 6 of the WMO.

The sponsor (also) has an insurance which is in accordance with the legal requirements in the Netherlands (Article 7 WMO and the Measure regarding Compulsory Insurance for Clinical Research in Humans of 23th June 2003). This insurance provides cover for damage to research subjects through injury or death caused by the study.

1. € 450.000,-- (i.e. four hundred and fifty thousand Euro) for death or injury for each subject who participates in the Research;
2. € 3.500.000,-- (i.e. three million five hundred thousand Euro) for death or injury for all subjects who participate in the Research;
3. € 5.000.000,-- (i.e. five million Euro) for the total damage incurred by the organisation for all damage disclosed by scientific research for the Sponsor as ‘verrichter’ in the meaning of said Act in each year of insurance coverage.

The insurance applies to the damage that becomes apparent during the study or within 4 years after the end of the study.

## Incentives (if applicable)

In case of travel to the LUMC, patients will be compensated for travel costs.

# ADMINISTRATIVE ASPECTS AND PUBLICATION

## Handling and storage of data and documents

A database for clinical characteristics and immunological testing will be established. At the moment a ProMISe databse for the registration of myasthenic patients is being developed. All data will be handled confidentially. All included subjects will be coded (LEMS +number in order of inclusion, e.g. LEMS001). The identification code is kept separate and only the registered investigators have access to it. Data and documents will be stored until 15 years after publication. The handling of the personal data will be done in compliance with the study protocol and the Dutch Personal Data Protection Act.

## Amendments

No amendments have been made.

## Annual progress report

The investigator will submit a summary of the progress of the trial to the accredited METC once a year. Information will be provided on the date of inclusion of the first subject, numbers of subjects included, other problems, and amendments.

## End of study report

The investigator will notify the accredited METC of the end of the study within a period of 8 weeks. The end of the study is defined as the end of follow-up period of the last patient (3 years after inclusion for determining tumour association and survival).

In case the study is ended prematurely, the investigator will notify the accredited METC within 15 days, including the reasons for the premature termination.

 Within one year after the end of the study, the investigator/sponsor will submit a final study report with the results of the study, including any publications/abstracts of the study, to the accredited METC.

## Public disclosure and publication policy

Results of this study will be sent without restrictions to international medical journals as well as medical conferences and/or patient associations in accordance to the CCMO statement on publication policy.

# REFERENCES

1. Titulaer MJ, Lang B, Verschuuren JJ. Lambert-Eaton myasthenic syndrome: from clinical characteristics to therapeutic strategies. Lancet Neurol 2011;10(12):1098-1107.

2. Wirtz PW, Sotodeh M, Nijnuis M et al. Difference in distribution of muscle weakness between myasthenia gravis and the Lambert-Eaton myasthenic syndrome. J Neurol Neurosurg Psychiatry 2002;73(6):766-768.

3. Titulaer MJ, Wirtz PW, Willems LN, van Kralingen KW, Smitt PA, Verschuuren JJ. Screening for small-cell lung cancer: a follow-up study of patients with Lambert-Eaton myasthenic syndrome. J Clin Oncol 2008;26(26):4276-4281.

4. Roberts A, Perera S, Lang B, Vincent A, Newsom-Davis J. Paraneoplastic myasthenic syndrome IgG inhibits 45Ca2+ flux in a human small cell carcinoma line. Nature 1985;317(6039):737-739.

5. Benatar M, Blaes F, Johnston I et al. Presynaptic neuronal antigens expressed by a small cell lung carcinoma cell line. J Neuroimmunol 2001;113(1):153-162.

6. Govindan R, Page N, Morgensztern D et al. Changing epidemiology of small-cell lung cancer in the United States over the last 30 years: analysis of the surveillance, epidemiologic, and end results database. J Clin Oncol 2006;24(28):4539-4544.

7. Paesmans M, Sculier JP, Lecomte J et al. Prognostic factors for patients with small cell lung carcinoma: analysis of a series of 763 patients included in 4 consecutive prospective trials with a minimum follow-up of 5 years. Cancer 2000;89(3):523-533.

8. Tammemagi CM, Neslund-Dudas C, Simoff M, Kvale P. Smoking and lung cancer survival: the role of comorbidity and treatment. Chest 2004;125(1):27-37.

9. Darnell RB, DeAngelis LM. Regression of small-cell lung carcinoma in patients with paraneoplastic neuronal antibodies. Lancet 1993;341(8836):21-22.

10. Zaheer W, Friedland ML, Cooper EB et al. Spontaneous regression of small cell carcinoma of lung associated with severe neuropathy. Cancer Invest 1993;11(3):306-309.

11. Wirtz PW, Lang B, Graus F et al. P/Q-type calcium channel antibodies, Lambert-Eaton myasthenic syndrome and survival in small cell lung cancer. J Neuroimmunol 2005;164(1-2):161-165.

12. Maddison P, Lang B. Paraneoplastic neurological autoimmunity and survival in small-cell lung cancer. J Neuroimmunol 2008;201-202:159-162.

13. Maddison P, Newsom-Davis J, Mills KR, Souhami RL. Favourable prognosis in Lambert-Eaton myasthenic syndrome and small-cell lung carcinoma. Lancet 1999;353(9147):117-118.

14. Titulaer MJ, Verschuuren JJ. Lambert-Eaton myasthenic syndrome: tumor versus nontumor forms. Ann N Y Acad Sci 2008;1132:129-134.

15. Eerola AK, Soini Y, Paakko P. A high number of tumor-infiltrating lymphocytes are associated with a small tumor size, low tumor stage, and a favorable prognosis in operated small cell lung carcinoma. Clin Cancer Res 2000;6(5):1875-1881.

16. Morris CS, Esiri MM, Marx A, Newsom-Davis J. Immunocytochemical characteristics of small cell lung carcinoma associated with the Lambert-Eaton myasthenic syndrome. Am J Pathol 1992;140(4):839-845.

17. Nelson BH. CD20+ B cells: the other tumor-infiltrating lymphocytes. J Immunol 2010;185(9):4977-4982.

18. Tani T, Tanaka K, Idezuka J, Nishizawa M. Regulatory T cells in paraneoplastic neurological syndromes. J Neuroimmunol 2008;196(1-2):166-169.

19. Sun Z, Chen J, Aakre J et al. Genetic variation in glutathione metabolism and DNA repair genes predicts survival of small-cell lung cancer patients. Ann Oncol 2010;21(10):2011-2016.

20. Knoefel LF, Werle-Schneider G, Dally H et al. Polymorphisms in the apoptotic pathway gene BCL-2 and survival in small cell lung cancer. J Thorac Oncol 2011;6(1):183-189.

21. Al Omar S, Middleton D, Marshall E et al. Associations between genes for killer immunoglobulin-like receptors and their ligands in patients with solid tumors. Hum Immunol 2010;71(10):976-981.

22. de Graaf MT, de Beukelaar JW, Haasnoot GW et al. HLA-DQ2+ individuals are susceptible to Hu-Ab associated paraneoplastic neurological syndromes. J Neuroimmunol 2010;226(1-2):147-149.

23. van Sonderen A, Wirtz, .W., Verschuuren JJ, Titulaer MJ. Paraneoplastic syndromes of the neuromuscular junction: therapeutic options in myasthenia gravis, lambert-eaton myasthenic syndrome, and neuromyotonia. Curr Treat Options Neurol 2013;15(2):224-239.

24. Titulaer MJ, Klooster R, Potman M et al. SOX antibodies in small-cell lung cancer and Lambert-Eaton myasthenic syndrome: frequency and relation with survival. J Clin Oncol 2009;27(26):4260-4267.

25. Titulaer MJ, Maddison P, Sont JK et al. Clinical Dutch-English Lambert-Eaton Myasthenic syndrome (LEMS) tumor association prediction score accurately predicts small-cell lung cancer in the LEMS. J Clin Oncol 2011;29(7):902-908.

26. Verschuuren JJ, Dalmau J, Tunkel R et al. Antibodies against the calcium channel beta-subunit in Lambert-Eaton myasthenic syndrome. Neurology 1998;50(2):475-479.

27. Wirtz PW, Willcox N, van der Slik AR et al. HLA and smoking in prediction and prognosis of small cell lung cancer in autoimmune Lambert-Eaton myasthenic syndrome. J Neuroimmunol 2005;159(1-2):230-237.

28. Wirtz PW, van Dijk JG, van Doorn PA et al. The epidemiology of the Lambert-Eaton myasthenic syndrome in the Netherlands. Neurology 2004;63(2):397-398.

29. Wirtz PW, Wintzen AR, Verschuuren JJ. Lambert-Eaton myasthenic syndrome has a more progressive course in patients with lung cancer. Muscle Nerve 2005;32(2):226-229.

30. Titulaer MJ, Wirtz PW, Kuks JB et al. The Lambert-Eaton myasthenic syndrome 1988-2008: a clinical picture in 97 patients. J Neuroimmunol 2008;201-202:153-158.

31. Wirtz PW, Verschuuren JJ, van Dijk JG et al. Efficacy of 3,4-diaminopyridine and pyridostigmine in the treatment of Lambert-Eaton myasthenic syndrome: a randomized, double-blind, placebo-controlled, crossover study. Clin Pharmacol Ther 2009;86(1):44-48.

32. Sabater L, Titulaer M, Saiz A, Verschuuren J, Gure AO, Graus F. SOX1 antibodies are markers of paraneoplastic Lambert-Eaton myasthenic syndrome. Neurology 2008;70(12):924-928.

33. Lipka AF, Verschuuren JJ, Titulaer MJ. SOX1 antibodies in Lambert-Eaton myasthenic syndrome and screening for small cell lung carcinoma. Ann N Y Acad Sci 2012;1275:70-77.

34. van Dongen M, Savage ND, Jordanova ES et al. Anti-inflammatory M2 type macrophages characterize metastasized and tyrosine kinase inhibitor-treated gastrointestinal stromal tumors. Int J Cancer 2010;127(4):899-909.

35. Heusinkveld M, de Vos van Steenwijk PJ, Goedemans R et al. M2 macrophages induced by prostaglandin E2 and IL-6 from cervical carcinoma are switched to activated M1 macrophages by CD4+ Th1 cells. J Immunol 2011;187(3):1157-1165.

36. Pander J, Heusinkveld M, van der Straaten T et al. Activation of tumor-promoting type 2 macrophages by EGFR-targeting antibody cetuximab. Clin Cancer Res 2011;17(17):5668-5673.

37. Gottlin EB, Bentley RC, Campa MJ, Pisetsky DS, Herndon JE, Patz EF, Jr. The Association of Intratumoral Germinal Centers with early-stage non-small cell lung cancer. J Thorac Oncol 2011;6(10):1687-1690.

38. Monstad SE, Drivsholm L, Storstein A et al. Hu and voltage-gated calcium channel (VGCC) antibodies related to the prognosis of small-cell lung cancer. J Clin Oncol 2004;22(5):795-800.

39. Barry EL, Viglione MP, Kim YI, Froehner SC. Expression and antibody inhibition of P-type calcium channels in human small-cell lung carcinoma cells. J Neurosci 1995;15(1 Pt 1):274-283.

40. Nagel A, Engel AG, Lang B, Newsom-Davis J, Fukuoka T. Lambert-Eaton myasthenic syndrome IgG depletes presynaptic membrane active zone particles by antigenic modulation. Ann Neurol 1988;24(4):552-558.

41. van Meerten T, van Rijn RS, Hol S, Hagenbeek A, Ebeling SB. Complement-induced cell death by rituximab depends on CD20 expression level and acts complementary to antibody-dependent cellular cytotoxicity. Clin Cancer Res 2006;12(13):4027-4035.

42. Cappello S, Liu NX, Musselli C, Brezicka FT, Livingston PO, Ragupathi G. Immunization of mice with fucosyl-GM1 conjugated with keyhole limpet hemocyanin results in antibodies against human small-cell lung cancer cells. Cancer Immunol Immunother 1999;48(9):483-492.

43. Parajuli P, Yanagawa H, Hanibuchi M et al. Humanized anti-ganglioside GM2 antibody is effective to induce antibody-dependent cell-mediated cytotoxicity in mononuclear cells from lung cancer patients. Cancer Lett 2001;165(2):179-184.

44. van der Burg SH, Piersma SJ, de JA et al. Association of cervical cancer with the presence of CD4+ regulatory T cells specific for human papillomavirus antigens. Proc Natl Acad Sci U S A 2007;104(29):12087-12092.

**UK IRB approved protocol**

A study of seronegative Lambert-Eaton myasthenic syndrome

Paul Maddison

Version 2.4 (March 2010)

**Sponsor:** Nottingham University Hospitals NHS Trust

**Chief Investigator:** Dr Paul Maddison

Consultant Neurologist

Nottingham University Hospitals NHS Trust

Queen’s Medical Centre

Nottingham

NG7 2UH

Tel: 0115 9249924 extension 66281

email: [paul.maddison@nhs.net](mailto:paul.maddison@nhs.net)

# Introduction and rationale

All patients with Lambert-Eaton myasthenic syndrome (LEMS) have an autoimmune antibody-mediated disorder, but the putative antibody (to voltage-gated calcium channels) is only present in 65-85% of patients. In this current study, we intend to identify and classify patients with LEMS who do not harbour typical P/Q-type voltage-gated calcium channel (VGCC) antibodies (seronegative LEMS), to produce a refined cohort of patients. A control group of seropositive LEMS patients will also be asked to participate. From this, further studies will be performed to search for candidate antigens in these seronegative LEMS patients. We would hope to establish the antibody target in these patients and ultimately provide a new antibody diagnostic test for all seronegative LEMS patients.

# Study endpoints

This is an observational study in which all UK LEMS patients will be invited to participate to characterise their neurological symptoms and signs and obtain a blood sample for further analysis.

## Study synopsis

Lambert-Eaton myasthenic syndrome is an autoimmune presynaptic disorder of neuromuscular transmission, characterised by impaired quantal release that causes fatiguable proximal muscle weakness (Lambert *et al.*, 1956, 1961; Rooke *et al.*, 1960; Lambert and Rooke, 1965; Elmqvist and Lambert, 1968; Lambert and Elmqvist, 1971) , depressed tendon reflexes and autonomic dysfunction (Rubenstein *et al*., 1979; Heath *et al*., 1988; Khurana *et al*., 1988; Waterman *et al.*, 1997; Suilleabhain *et al*., 1998). Approximately 60% of patients with LEMS have associated small-cell lung cancer (SCLC) (O’Neill *et al*., 1988).

In LEMS, IgG antibodies to presynaptic P/Q-type voltage-gated calcium channel antibodies cause muscle weakness and autonomic dysfunction by interfering with Ca^2+^ -dependent neurotransmitter release. Autoantibodies directed against P/Q-type VGCCs are detectable in about 85% of all LEMS patients (Motomura *et al*., 1995; Lennon *et al*., 1995): the antigenic target in the 15% of remaining cases is unknown. It is thought that the antigenic stimulus for anti-VGCC autoantibody production in patients with SCLC and associated LEMS is from the tumour’s own VGCCs. Anti-VGCC antibodies that seem to be provoked by the cancer can block Ca^2+^ influx in SCLC cell lines, which suggest that in vivo they may inhibit tumour growth. However, the triggering factor in autoimmune (non-cancer-associated) LEMS is unknown.

Although almost all patients with SCLC and LEMS have detectable VGCC antibodies, in autoimmune (non-cancer-associated) LEMS only about 65-75% of cases have VGCC antibodies (Nakao et al, 2002). Many of these seronegative patients show beneficial responses to immunomodulatory therapy and animal studies have shown that similar nerve-muscle junction defects can be transferred to mice by both seronegative and seropositive LEMS patients (Nakao et al, 2002). Although Takamori et al (2000) showed that some seronegative LEMS patients had antibodies to the presynaptic protein synaptotagmin-1, these antibodies were not specific to seronegative LEMS patients and were seen in a similar number of LEMS patients with VGCC antibodies. Additionally, passive transfer of sera from synaptotagmin-positive patients to mice did not provoke the characteristic pattern of weakness seen following transfer of LEMS IgG (Takamori et al, 1994). There have been no studies performed since to replicate these findings, or to determine the nature of other antibodies that may cause seronegative LEMS.

# Patient population

All UK patients with LEMS.

# Methods

In collaboration with groups in Holland and Japan, we are currently collecting a cohort of well-characterised seronegative LEMS patients. We will register with the British Neurological Surveillance Unit (BNSU) of the Association of British Neurologists (P. Maddison, Ordinary Member) to allow patients with LEMS identified by other UK neurologists to be enrolled on a monthly basis into this study, and reviewed by us.

The LEMS patients would be recruited and studied in the following manner:

1. Patients would be classified as suffering from LEMS if they exhibited classical symptoms and signs of this disorder and characteristic neurophysiological abnormalities.
   1. Classical symptoms and signs would include (O’Neill *et al.*, 1988):

Initial fatiguable proximal lower limb weakness (expected symptom and sign in 90% of patients).

Autonomic dysfunction (dry mouth, blurred vision, erectile dysfunction etc) (expected symptom in 80%).

Attenuated or absent tendon reflexes with or without reflex potentiation following 10 s maximal voluntary contraction (expected sign in 92%).

Lack of significant sensory abnormalities (expected symptom and sign in 96%).

- 1. Classical neurophysiological findings would include (Maddison *et al*., 1998; Oh *et al*., 2005; Oh *et al*., 2007):

Small resting compound muscle action potential amplitudes (CMAPs) in either abductor digiti minimi (ADM), or abductor pollicis brevis (APB) (expected in 90% of patients).

Decremental CMAP responses (>8%) at low rates (3 Hz) repetitive nerve stimulation in ADM (expected in 87-94%).

Incremental CMAP responses (>60%) at either high rate (>20 Hz) repetitive nerve stimulation or after 10 s maximal voluntary activity in ADM or APB (expected in 90-97%).

Possible additional evidence of a disorder of neuromuscular transmission from single fibre electromyography (expected in 100%).

1. Typically, patients will have normal creatine kinase (CK), thyroid function and inflammatory marker (CRP, ESR) blood tests, and often exhibit an autoimmune diathesis.

Patients with the above symptoms, signs, and neurophysiological abnormalities would be identified through normal daily practice both locally by the lead investigator (P. Maddison), and elsewhere by other UK Neurologists.

As a matter of routine diagnostic investigation, neurologists suspecting LEMS send blood to Dr Bethan Lang at the department of Neurosciences at the Weatherall Institute of Molecular Medicine at the John Radcliffe Hospital, Oxford, for analysis of P/Q-type VGCC antibodies. This is a highly specific test for LEMS and when it is known that a patient tests positive for P/Q-type VGCC antibodies, they will be classed as seropositive LEMS for potential recruitment into the control arm of this study.

The study lead (Dr P Maddison) will be informed of the existence of seropositive LEMS patients by the laboratory in Oxford. Dr Maddison will also be informed of the existence of both seropositive and seronegative LEMS patients by the treating UK Neurologist through the BNSU. Dr Maddison or his research fellow will then contact the neurologist in charge of the LEMS patient’s care. The local Neurologist will have to do no more than inform the LEMS patient under their care that there is an ongoing study relating to improving the diagnosis in seronegative LEMS.

The identified patients will be sent an information letter outlining the nature of the study, and invited to take part (via email, telephone call, or letter). If the patient agrees to participate in the study, they fill out a personal details and consent form and send it to us via a stamped addressed envelope.

For all study participants, arrangements will be made to review the patient. The patient will be offered assessment either in their own home, or alternatively at Queen’s Medical Centre, Nottingham (to see P. Maddison in his Muscle / Myasthenia clinic), if the patient is willing to travel.

During the assessment, signed consent will be obtained from the patient. A single blood sample will be taken, and the patient’s neurological symptoms and signs will be assessed. The assessment will take approximately 40 minutes. Their uninterrupted care would continue under Dr Maddison, or the referring Neurologist.

The whole blood sample (marked anonymously) and the spun serum sample will be frozen at –20oC initially, and then transferred to –80oC for storage, within the freezer of the Department of Neurology at Queen’s Medical Centre, Nottingham. When between 10-20 patients have been included in the study, over a 3 year period, the samples will be transferred to Dr Bethan Lang, at the department of Neurosciences, Weatherall Institute of Molecular Medicine, John Radcliffe Hospital, Oxford, for further immunoprecipitation, immunohistochemical, and cellular expression studies.

We will use standard immunoprecipitation techniques to look for antibodies to other VGCCs, including the β and γδ subunits and N and L-type subtypes. Our intention to identify and classify seronegative LEMS patients would be the first such study and would provide a refined cohort from which further studies could be performed when suitable diagnostic tests for alternative candidate antigens become available. Ultimately, we would hope to establish a diagnostic test for seronegative-LEMS. Autoantibodies present only in seronegative autoimmune LEMS would reassure patients (and clinicians) that their LEMS symptoms were unlikely to be due to the presence of an underlying lung tumour.

# Participating centres

All patients will be offered a choice between being seen in their own home, or alternatively by travelling to visit the lead investigator, Dr P. Maddison, for review in his well-established muscle / myasthenia clinic at Queen’s Medical Centre, Nottingham.

# Patient confidentiality

All patients’ study records and blood samples will be anonymised, and only the principal investigator (P. Maddison) will hold separate patient contact information relating to the study codes, in a locked hospital office. The stored blood samples will be kept in a locked freezer within the Department of Neurology, Nottingham University Hospitals. The freezer is licensed under the tissue holding act, and only Dr Maddison will have access to these blood samples.

# Use of information / publication

All published results will be available to the study patients and all referring Neurologists via scientific publications, and reports issued to the Myasthenia Gravis Association.

# Anticipated trial duration

For each individual patient, the study will only involve one single visit, with full clinical evaluation only lasting about one hour. The study would hope to recruit up to 18 seronegative LEMS patients over a three year period, and approximately 100 seropositive LEMS cases. We have already identified about 6 patients with seronegative LEMS after informal discussions with members of the myasthenia interest group of the Association of British Neurologists, after about 12 months.

This study will in no way impact on the normal ongoing management of each patient. The planned research will provide us with a unique cohort of patients with seronegative LEMS. Hopefully, we would aim to identify the putative antibody responsible for the symptoms and signs of seronegative LEMS, and to eventually provide a diagnostic assay for all LEMS patients.

# References

Elmqvist D, Lambert EH. Detailed analysis of neuromuscular transmission in a patient with the myasthenic syndrome sometimes associated with bronchogenic carcinoma. Mayo Clinic Proceedings 1968; 43: 689-713.

Heath JP, Ewing DJ, Cull RE. Abnormalities of autonomic function in the Lambert-Eaton myasthenic syndrome. Journal of Neurology Neurosurgery and Psychiatry 1988; 51: 436-9.

Khurana RK, Koski CL, Mayer RF. Autonomic dysfunction in Lambert-Eaton myasthenic syndrome. Journal of the Neurological Sciences 1988; 85: 77-86.

Lambert EH, Rooke ED. Myasthenic state and lung cancer. In: Brain WR, Norris FH, editors. The remote effects of cancer on the nervous system. New York and London: Grune and Stratton; 1965. p. 67-80.

Lambert EH, Elmqvist D. Quantal components of end-plate potentials in the myasthenic syndrome. Annals of the New York Academy of Sciences 1971; 183: 183-99.

Lambert EH, Eaton LM, Rooke ED. Defect of neuromuscular conduction associated with malignant neoplasms. American Journal of Physiology 1956; 187: 612-3.

Lambert EH, Rooke ED, Eaton LM, Hodgson CH. Myasthenic syndrome occasionally associated with bronchial neoplasm: neurophysiologic studies. In: Viets HR, editor. Myasthenia gravis. Springfield, Illinois: CC Thomas; 1961. p. 362-410.

Lang B, Waterman S, Pinto A, Jones D, Moss F, Boot J, Brust P, Williams M, Stauderman K, Harpold M, Motomura M, Moll JW, Vincent A, Newsom-Davis J. The role of autoantibodies in Lambert-Eaton myasthenic syndrome. Annals of the New York Academy of Sciences 1998; 841: 596-605.

Lennon VA, Kryzer TJ, Griesmann GE, O’Suilleabhain PE, Windebank AJ, Woppmann A, Miljanich GP, Lambert EH. Calcium-channel antibodies in the Lambert-Eaton syndrome and other paraneoplastic syndromes. New England Journal of Medicine 1995; 332: 1467-74.

Maddison P, Newsom-Davis J, Mills KR. Distribution of electrophysiological abnormality in Lambert-Eaton myasthenic syndrome. J Neurol Neurosurg Psychiatry 1998; 65: 213-7.

Motomura M, Johnston I, Lang B, Vincent A, Newsom-Davis J. An improved diagnostic assay for Lambert-Eaton myasthenic syndrome. Journal of Neurology Neurosurgery and Psychiatry 1995; 58: 85-7.

Nakao YK, Motomura M, Fukudome T, Fukuda T, Shiraishi H, Yoshimura T, Tsujihata M, Eguchi K. Seronegative Lambert-Eaton myasthenic syndrome: study of 110 Japanese patients. Neurology 2002; 59: 1773-5.

Oh SJ, Kurokawa K, Claussen GC, Ryan HF Jr. Electrophysiological diagnostic criteria of Lambert-Eaton myasthenic syndrome. Muscle Nerve 2005; 32: 515-20.

Oh SJ, Hatanaka Y, Claussen GC, Sher E. Electrophysiological differences in seropositive and seronegative Lambert-Eaton myasthenic syndrome. Muscle Nerve 2007; 35: 178-83.

O’Neill JH, Murray NMF, Newsom-Davis. The Lambert-Eaton myasthenic syndrome: a review of 50 cases. Brain 1988; 111: 577-96.

Rooke ED, Eaton LM, Lambert EH, Hodgson CH. Myasthenia and malignant intrathoracic tumor. The Medical Clinics of North America 1960; 44: 977-88.

Rubenstein AE, Horowitz SH, Bender AN. Cholinergic dysautonomia and Eaton-Lambert syndrome. Neurology 1979; 29: 720-3.

Suilleabhain P, Low PA, Lennon VA. Autonomic dysfunction in the Lambert-Eaton myasthenic syndrome: serologic and clinical correlates. Neurology 1998; 50: 88-93.

Takamori M, Hamada T, Komai K, Takahashi M, Yoshida A. Synaptotagmin can cause an immune-mediated model of Lambert-Eaton myasthenic syndrome in rats. Annals of Neurology 1994; 35: 74-80.

Takamori M, Komai K, Iwasa K. Antibodies to calcium channel and synaptotagmin in Lambert-Eaton myasthenic syndrome. The American Journal of the Medical Sciences 2000; 319: 204-8.

Waterman SA, Lang B, Newsom-Davis J. Effect of Lambert-Eaton myasthenic syndrome antibodies on autonomic neurons in the mouse. Annals of Neurology 1997; 42: 147-56.
